# Serial-Multiple Mediation of Job Burnout and Fatigue in the Relationship Between Sickness Presenteeism and Productivity Loss in Nurses: A Multicenter Cross-Sectional Study

**DOI:** 10.3389/fpubh.2021.812737

**Published:** 2022-01-14

**Authors:** Yuxin Li, Bingmei Guo, Yongchao Wang, Xiaoyan Lv, Rong Li, Xiangyun Guan, Li Li, Junli Li, Yingjuan Cao

**Affiliations:** ^1^School of Nursing and Rehabilitation, Cheeloo College of Medicine, Shandong University, Jinan, China; ^2^Department of Nursing, Qilu Hospital, Cheeloo College of Medicine, Shandong University, Jinan, China; ^3^Nursing Theory and Practice Innovation Research Center, Cheeloo College of Medicine, Shandong University, Jinan, China; ^4^Department of Biostatistics, School of Public Health, Cheeloo College of Medicine, Shandong University, Jinan, China; ^5^Institute for Medical Dataology, Shandong University, Jinan, China

**Keywords:** China, nurses, presenteeism, burnout, productivity, fatigue, cross-sectional studies, mediation analysis

## Abstract

**Background:**

In China, sickness presenteeism, job burnout, and fatigue are common among nurses during the COVID-19 pandemic. We propose the prevalence of sickness presenteeism can adversely affect nurses' physical and mental health, negatively impact their work productivity and quality, and pose a threat to patients' safety. Therefore, this study examines the mechanism of productivity loss caused by sickness presenteeism, fatigue, and job burnout.

**Objectives:**

To investigate the serial-multiple mediating effect of job burnout and fatigue in the relationship between sickness presenteeism and productivity loss among nurses.

**Methods:**

A multicenter cross-sectional survey was undertaken by administering an online questionnaire from December 2020 to May 2021. Stratified cluster sampling was used to include 3,491 nurses from 14 hospitals in Shandong Province, China. Variables were measured using the Sickness Presenteeism Questionnaire, Stanford Presenteeism Scale, Chalder Fatigue Scale, and Maslach Burnout Inventory. Data analyses were carried out using descriptive statistics, one-way analysis of variance, independent-samples *t*-test, Pearson correlation analysis, hierarchical regression, and bootstrapping method.

**Results:**

From the 3,491 nurses who volunteered in this online survey, only 2,968 valid questionnaires were returned. Sickness presenteeism exhibited a prevalence of 70.6% during the COVID-19 pandemic. The average score of health-related productivity loss was 15.05 ± 4.52, fatigue was 8.48 ± 3.40, and job burnout was 39.14 ± 19.64. Sickness presenteeism was positively associated with fatigue and job burnout while job burnout was positively associated with nurse fatigue. Sickness presenteeism, fatigue, and job burnout were also positively correlated with health-related productivity loss. Statistically significant paths via the single mediation of fatigue and job burnout were established. A statistically significant serial-multiple mediating effect of fatigue and job burnout on the association between sickness presenteeism and productivity loss accounted for 35.12% of the total effect size.

**Conclusions:**

There was a high incidence of sickness presenteeism and job burnout among Chinese nurses. High-frequency sickness presenteeism may result in increased productivity loss through the two mediating effects of fatigue and job burnout. Sickness presenteeism may increase fatigue, promote job burnout, and result in increased productivity loss among Chinese nurses during the COVID-19 pandemic.

## Introduction

The COVID-19 pandemic has resulted in the increased vulnerability of medical workers to job burnout and fatigue because of the escalating number of patients, emotional loss of colleagues, and risk of infection (1). The lack of intervention to address these issues could have long-term risks for staff and patients. Accordingly, institutions should focus on the determinants of job burnout and fatigue and explore their effects on health workers. Job burnout is defined by the three dimensions of exhaustion, cynicism, and inefficacy and is a prolonged response to chronic stressors on the job (2). Studies have found job burnout to be related to job performance and health-related productivity so it can significantly impact both employees and the organization (2). Fatigue begins gradually, can last more than 6 months, and is relieved only by rest (3). Studies have shown fatigue can be divided into physical fatigue and mental fatigue (4).

Despite suffering from physical and mental symptoms such as job burnout and fatigue, nurses have continued to work during this crisis due to feelings of responsibility and professionalism (5). Most scholars define the situation of people who continue to work while in an unhealthy state as sickness presenteeism (6–8). However, sickness presenteeism behaviors are considered a symbol of traditional dedication and diligence in eastern cultures and are perceived as meritorious service by Chinese mainstream media. Recently, scholars have began examining the work-related death of medical staff and explored the role of sickness presenteeism, overwork, and exhaustion (8–10). While the overall level of sickness absence has recently declined, this does not portend a healthier working population since there is a corresponding increase in overall sickness presenteeism (11). Evidence suggests that nurses are a susceptible group for sickness presenteeism (12). According to a review by Freeling et al., the prevalence of sickness presenteeism in nurses ranged from 15.7 to 87.0% (13).

Sickness presenteeism behaviors among nurses tend to reduce patients' quality of care and negatively affect their treatment, rehabilitation, and safety (14). Sickness presenteeism behaviors may also negatively affect nurses mental and physical health, reduce their work engagement and job satisfaction, increase job burnout (15), induce future health issues, enhance the chance of long-term absenteeism, and impose direct and indirect financial burdens on healthcare organizations (16). Moreover, while previous research has linked sickness presenteeism with job performance or productivity, the mechanism of psychological burnout and physical fatigue in this relationship has not been explored in detail (7, 17–19). Nurses play an important role in universal healthcare, thus clarifying the mechanisms and links between nurse sickness presenteeism and health-related productivity loss is important (13). Consequently, paying attention to occupational health of nurses has profound implications for promoting public health and improving the overall quality of health care in the country (20). Although the mechanisms and links in the fields of occupational health and human resource management have attracted the wide attention of scholars, these related studies were mainly conducted in the USA and European countries, but rarely in the context of Chinese culture (13).

Studies found that employee job burnout was closely associated with sickness presenteeism and caused loss of productivity (21–23). In addition, Aboagye et al. proved that sickness presenteeism independently enhanced the risks of moderate or severe fatigue and worsened work performance in the past year (17). Fatigue was also strongly associated with lost productivity (17). Physical and mental fatigue were positively correlated with job burnout as highlighted in a previous study (24). Therefore, we hypothesized that some factors might mediate between sickness presenteeism and a loss of productivity. Therefore, the hypotheses of this study are as follows: (i) sickness presenteeism, fatigue, and job burnout have different effects on productivity loss, (ii) fatigue acts as a moderator between sickness presenteeism and productivity loss, (iii) job burnout acts as a moderator between sickness presenteeism and productivity loss, and (iv) fatigue and job burnout have serial-multiple mediating effect between sickness presenteeism and productivity loss. This study aims to investigate the serial-multiple mediating effect of job burnout and fatigue in the relationship between sickness presenteeism and productivity loss among nurses. The study also aims to provide a theoretical basis for alleviating significant productivity loss among these health care professionals.

## Materials and Methods

### Design, Setting, and Participants

This multicenter cross-sectional research used data from the first survey of Chinese nurses conducted December 2020 to May 2021 from the Nurses' Health Cohort Study of Shandong (registration number: ChiCTR2100043202). A multistage sampling method was adopted as follows: (a) Shandong Province was divided geographically into eastern, western, southern, northern, and central regions, (b) at least two hospitals in each region were selected by convenience sampling, and (c) all nurses at selected hospitals received invitations and were recruited (cluster sampling).

Inclusion criteria were registered nurses with nurse qualification certificates and who volunteered. Exclusion criteria were (a) retired, refresher, and student nurses, (b) nurses who suffered from severe mental illness or took psychotropic drugs; (c) nurses who had been working for <6 months; and (d) nurses who were on leave during the investigation.

### Measures

#### Demographic Variables

Demographic characteristics included gender, age, marital status, education level, department, employment type, professional title, position, and monthly income.

#### Sickness Presenteeism

The Sickness Presenteeism Questionnaire developed by Aronsson includes a single item “Has it happened over the previous 12 months that you have gone to work despite feeling that you really should have taken sick leave due to your state of health?” Responses are evaluated on a 4-point scale: never (1 point), once (2 points), two to five times (3 points), and >5 times (4 points). A higher score indicates a higher frequency of working while sick (6). Hou et al. conducted cross-cultural adaptation and validation of the questionnaire among Chinese medical professionals (25). The Sickness Presenteeism Questionnaire has been extensively used in the measurement of sickness presenteeism of Chinese medical staff (26–28).

#### Health-Related Productivity Loss

Productivity loss due to health issues was evaluated with the 6-item Stanford Presenteeism Scale (29), translated into Chinese by Zhao et al. (30). The Cronbach's alpha coefficient was 0.76–0.90 and the scale is extensively used in the measurement of work productivity activity. The scale contains two dimensions of work energy and work limits. A 5-point Likert scale is adopted to score each item: completely disagree (1 point), totally agree (5 points). The total score ranges from 6 to 30 points. A higher score from the scale means a greater loss of productivity due to health issues brought about by sickness presenteeism among nurses. In the present study, the Cronbach's alpha coefficient of the scale was 0.795.

#### Job Burnout

Job burnout was assessed by the 22-item Maslach Burnout Inventory (31), which has three dimensions of low personal accomplishment, depersonalization, and emotional exhaustion. The Chinese version of the Maslach Burnout Inventory exhibited sufficient validity and reliability in Chinese nurses. The Cronbach's alpha coefficient was 0.788 and the re-test reliability coefficient was 0.713 (32). A 7-point Likert scale is adopted to rate each item, with completely inconsistent (1 point) and completely consistent (7 points). A higher score represents more severe job burnout. The Cronbach's alpha coefficient of the Chinese version of the Maslach Burnout Inventory in this study was 0.896.

#### Fatigue

The 14-item Chalder Fatigue Scale developed by T Chalder determines the severity of fatigue (33). The scale was translated into a Chinese version by Wang et al. (34). The Cronbach's alpha coefficient was 0.773 and the re-test reliability coefficient was 0.745 (34), which was found to be valid, and culturally sensitive (35). The scale consists of 14 “yes” (1 point) or “no” (0 point) questions, which are divided into two dimensions: physical fatigue and mental fatigue. A higher score from this scale suggests a higher level of fatigue. The Cronbach's alpha coefficient of this scale in this study was 0.803.

### Data Collection

Trained nurses with a master's degree and a professional background in medicine served as researchers. Permission was attained from the head of each nursing administrative department before the start of the investigation. All heads were subject to research-related training on the background, aim, and study methods. All queries were clarified in detail. Liaisons were set in test hospitals and functioned as regulators to ensure questionnaire completion and communicate with respondents on unresolved problems. Since the survey was web-based, the invitation for participation was disseminated in the form of a two-dimension code. Questionnaires were distributed via WeChat (a widely used instant messaging app used in China) in batches to allow respondents the opportunity to be relaxed, forthcoming, and detailed when responding to questions. We anticipated each of the eight questionnaires would be completed in approximately 10–15 min. Only professionals who signed non-disclosure agreements had direct access to the data. To ensure data quality, we adopted the required questions and data logic control design for the electronic questionnaires.

### Ethical Considerations

Participation was voluntary, and nurses provided written informed consent. The study was approved by the Shandong University Qilu Hospital Medical Ethics Committee (Registration number KYLL-202011-085).

### Data Analysis

Statistical analyses were carried out using R software version 4.0.5 (R Development Core Team, Vienna, Austria). The steps for analysis were as follows: (1) Descriptive statistics were applied on continuous variables mean and standard deviation (SD) and categorical variables (frequency and percentage). One-way analysis of variance (ANOVA) and independent-samples *t*-test compared sickness presenteeism, job burnout, fatigue, and health-related productivity loss from nurses with varying demographic variables from different groups. Bonferroni correction was applied for correcting the multiple test and *p* value < 0.006 was considered statistically significant for the univariate analysis. (2) We adopted a Pearson correlation analysis to examine the associations between variables. (3) We verified the important serial-multiple mediating effect of fatigue and job burnout on the relationship between sickness presenteeism and health-related productivity loss. We adopted bootstrap method that bias-corrected 95% confidence intervals (CIs) estimated based on 5,000 bootstrapped samples from the Serial-Multiple Mediation Model 6 of PROCESS macro version 3.5 in R produced by Hayes (36). In the analysis, all variables were incorporated into the tested model and the unstandardized coefficients of paths were generated to decrease Type 1 errors caused by data distribution (36). (4) We considered health-related productivity loss a dependent variable and performed a hierarchical linear regression analysis of variables to determine the variation of sickness presenteeism, fatigue, and job burnout for the regression equation. The demographic variables were included in the first layer of linear regression analysis (Model 1). Additionally, sickness presenteeism as an independent variable was entered into the second layer of linear regression analysis (Model 2). Finally, the mediating variable fatigue was entered the third layer as a new variable (Model 3) and then the mediating variable job burnout was entered the fourth layer as a new variable (Model 4). According to Bonferroni correction, *p* value < 0.013 was considered statistically significant for the regression analysis.

## Results

### Nurses' Demographic Characteristics Related to Sickness Presenteeism, Fatigue, Job Burnout, and Health-Related Productivity Loss

The present study involved a total of 3,491 nurses from 14 hospitals in Shandong Province, China. Questionnaires that were incomplete or logically inconsistent were eliminated, which led to the exclusion of 15% (*n* = 523) respondents. Of the remaining 2,968 participants, approximately 88.27% were clinical nurses and 95.38% were female. Table 1 summarizes the descriptive statistics of the participants' sickness presenteeism scores, of which the average score was 2.19 (*SD* = 0.97). Specifically, 70.6% (2,095) of the participants had sickness presenteeism behaviors. The demographic characteristics associated with nurses' sickness presenteeism are shown in Supplementary Table 1. The results indicated that the average score of health-related productivity loss was 15.05 (*SD* = 4.52), the average score of fatigue was 8.48 (*SD* = 3.40), and the average score of job burnout was 39.14 (*SD* = 19.64). Descriptives of demographic characteristics associated with nurses' fatigue or productivity loss are shown in Supplementary Tables 2, 3. Table 2 shows respondents' demographic characteristics and univariate analysis of job burnout.

**Table 1:** Descriptive statistics of the nurses' sickness presenteeism (*N* = 2,968).

**Item**	**Never (*n*, %)**	**Once (*n*, %)**	**2–5 times (*n*, %)**	**>5 times (*n*, %)**
Has it happened over the previous 12 months that you have gone to work despite feeling that you really should have taken sick leave due to your state of health	873 (29.4)	939 (31.6)	867 (29.2)	289 (9.7)

**Table 2:** Respondent characteristics and univariate analysis of demographic factors related to job burnout in nurses.

**Variables**	***n* (%)**	**Job burnout (x ±SD)**	**t/F value^†^**	***p*-value**
Total	2,968 (100.00)	39.14 ± 19.64		
**Gender**				
Male	137 (4.62)	47.30 ± 19.50	**24.999**	**<0.001** ^*^
Female	2,831 (95.38)	38.75 ± 19.56		
**Age, years**				
<30	745 (25.10)	43.52 ± 19.99	**34.419**	**<0.001** ^*^
30–39	1,652 (55.66)	39.38 ± 19.03		
40–49	460 (15.50)	33.33 ± 19.73		
≥50	111 (3.74)	30.34 ± 16.87		
**Marital status**				
Unmarried	637 (21.46)	43.77 ± 19.76	**15.670**	**<0.001** ^*^
Married	2,273 (76.58)	37.95 ± 19.43		
Divorced	39 (1.31)	35.74 ± 17.17		
Others	19 (0.64)	33.95 ± 21.78		
**Education^‡^**				
Secondary vocational degree	789 (26.58)	35.80 ± 18.75	**12.262**	**0.001** ^*^
Associate's degree	1,613 (54.35)	39.97 ± 20.00		
Bachelor's degree	557 (18.77)	41.25 ± 19.26		
Master's degree	9 (0.30)	52.67 ± 14.16		
**Professional title**				
Junior	1,579 (53.20)	41.58 ± 19.85	**23.232**	**<0.001** ^*^
Intermediate	1,198 (40.36)	37.21 ± 19.03		
Assistant senior	184 (6.20)	31.18 ± 18.18		
Senior	7 (0.24)	28.71 ± 17.42		
**Employment type**				
Permanent staff	886 (29.85)	36.79 ± 19.12	**8.254**	**<0.001** ^*^
Personnel agency	1,534 (51.68)	39.28 ± 19.57		
Contract staff	356 (11.99)	40.88 ± 19.62		
Labor dispatch	133 (4.48)	46.84 ± 20.60		
Filing staff	38 (1.28)	45.92 ± 22.54		
Others	21 (0.71)	37.14 ± 17.87		
**Department**				
Internal medicine	849 (28.61)	40.58 ± 19.36	**4.739**	**<0.001** ^*^
Surgery	624 (21.02)	39.04 ± 19.26		
Emergency	183 (6.17)	42.49 ± 19.89		
Gynecology	76 (2.56)	35.38 ± 19.43		
Obstetrics	144 (4.85)	33.67 ± 19.18		
Pediatrics	264 (8.89)	38.59 ± 19.91		
Operating room	235 (7.92)	39.80 ± 19.26		
ICU	175 (5.90)	41.13 ± 19.69		
Outpatient	87 (2.93)	30.00 ± 17.38		
Administration	6 (0.20)	29.33 ± 18.18		
Others	325 (10.95)	38.51 ± 20.63		
**Position**				
Clinical nurse	2,620 (88.27)	40.09 ± 19.61	**11.176**	**<0.001** ^*^
Deputy head nurse	150 (5.05)	32.13 ± 18.82		
Head nurse	185 (6.23)	32.15 ± 18.37		
General head nurse	4 (0.13)	41.75 ± 5.68		
Deputy director of nursing department	5 (0.17)	19.20 ± 8.82		
Director of nursing department	4 (0.13)	29.00 ± 16.51		
**Monthly income, CNY**				
<3,000	195 (6.57)	42.53 ± 20.82	**4.193**	**0.002** ^*^
3,000–5,999	1,504 (50.67)	39.76 ± 19.85		
6,000–8,999	944 (31.81)	38.34 ± 19.26		
9,000–19,999	280 (9.43)	37.26 ± 18.80		
≥12,000	45 (1.52)	39.14 ± 19.64		

*SD, standard deviation; ICU, intensive care unit; CNY, China Yuan*.

*Bold value for p < 0.05*.

* *Statistically significant differences in the variables after application of Bonferroni correction (p < 0.006)*.

† *One-way ANOVA was carried out for more than two groups, and independent-samples t-test was adopted for two groups*.

‡ *Secondary vocational degree: Having a 4-year senior high school study experience of professional training; associate's degree: Having a 3-year college study experience of professional training; bachelor's degree: Having a 4- or 5-year undergraduate course of training*.

According to the data, job burnout was significantly associated with gender as male nurses suffered greater job burnout than female nurses (*t* = 29.999, *p* < 0.001). Job burnout also varied by age (*F* = 34.419, *p* < 0.001) and marital status (*F* = 15.670, *p* < 0.001). Further *posthoc* analysis showed younger nurses experienced higher levels of job burnout, and unmarried nurses had higher levels of job burnout than married nurses. Additionally, nurses of different education backgrounds obtained significantly different job burnout scores (*F* = 12.262, *p* < 0.001). A *posthoc* analysis showed that job burnout of respondents with a higher educational level was significantly more severe. In addition, one-way ANOVA revealed significantly different degrees of job burnout between different employment types (*F* = 8.254, *p* < 0.001), professional titles (*F* = 23.232, *p* < 0.001), and positions (*F* = 11.176, *p* < 0.001). Further *posthoc* analysis showed that permanent staff nurses suffered lower levels of job burnout than other types of nurses employed. Conversely, nurses with lower professional titles experienced higher levels of job burnout, and clinical nurses suffered a higher level of job burnout than head nurses.

### Correlation Analysis

The statistics of means (SDs) and correlations between the four investigated variables, namely sickness presenteeism, fatigue, job burnout, and productivity loss, are summarized in Table 3. Consistent with our expectation, health-related productivity loss was positively correlated with the nurses' sickness presenteeism (*r* = 0.282, *p* < 0.001), fatigue (*r* = 0.392, *p* < 0.001), and job burnout (*r* = 0.488, *p* < 0.001). Sickness presenteeism was positively associated with fatigue (*r* = 0.201, *p* < 0.001) and job burnout (*r* = 0.164, *p* < 0.001). Job burnout was positively associated with nurses' fatigue (*r* = 0.447, *p* < 0.001).

**Table 3:** Correlations between sickness presenteeism, fatigue, job burnout and health-related productivity loss (*N* = 2,968).

**Variables**	**1**	**2**	**3**	**4**
1. Sickness presenteeism	1.000			
2. Fatigue	0.201^***^	1.000		
3. Job burnout	0.164^***^	0.447^***^	1.000	
4. Health-related productivity loss	0.282^***^	0.392^***^	0.488^***^	1.000
Mean	2.193	8.475	39.140	15.055
Standard deviation	0.969	3.401	19.635	4.524

*** *p < 0.001*.

*Covariates include age, gender, marital status, education, professional title, employment type, department, position, and monthly income*.

### Hierarchical Linear Regression Analysis of Variables Associated With Health-Related Productivity Loss

We then considered health-related productivity loss a dependent variable and performed hierarchical linear regression analysis of variables (Table 4). Model 1 (*R*^2^ = 0.026, *F* = 8.925, Δ*R*^2^ = 0.026, Δ*F* = 8.925, *p* < 0.001) included only demographic variables, showed that gender (B = −1.262, *p* = 0.002), age (B = −0.399, *p* = 0.026), and position (B = −0.617, *p* < 0.001) were statistically significantly associated with health-related productivity loss, respectively. Additionally, Model 2 (*R*^2^ = 0.104, *F* = 34.226, Δ*R*^2^ = 0.077, Δ*F* = 255.034, *p* < 0.001) included demographic variables and sickness presenteeism, and showed that health-related productivity loss was statistically significantly associated with sickness presenteeism (B = 1.307, *p* < 0.001), gender (B = −1.195, *p* = 0.002), department (B = −0.054, *p* = 0.022) and position (B = −0.702, *p* < 0.001), respectively. Model 3 (*R*^2^ = 0.218, *F* = 74.981, Δ*R*^2^ = 0.114, Δ*F* = 432.581, *p* < 0.001) included demographic variables, sickness presenteeism and fatigue, and showed that health-related productivity loss was statistically significantly associated with sickness presenteeism (B = 0.981, *p* < 0.001), fatigue (B = 0.461, *p* < 0.001), gender (B = −1.093, *p* = 0.002), and position (B = −0.470, *p* = 0.002), respectively. Finally, Model 4 (*R*^2^ = 0.326, *F* = 119.173, Δ*R*^2^ = 0.108, Δ*F* = 473.460, *p* < 0.001) included demographic variables, sickness presenteeism, fatigue, and job burnout, and showed that health-related productivity loss was statistically significantly associated with sickness presenteeism (B = 0.848, *p* < 0.001), fatigue (B = 0.248, *p* < 0.001), job burnout (B = 0.087, *p* < 0.001), and position (B = −0.398, *p* = 0.002), respectively. The results of Model 4 suggested that sickness presenteeism, fatigue and job burnout could explain 30.0% of the variation of the final regression equation (Δ*R*^2^ = 0.300, *p* < 0.001).

**Table 4:** Hierarchical linear regression analysis of variables related to health-related productivity loss (*N* = 2,968).

**Variables**	**Health-related productivity loss**			
	**Model 1 (coef)**	**Model 2 (coef)**	**Model 3 (coef)**	**Model 4 (coef)**
**Control variables**
Gender	**−1.262** ^*^	**−1.195** ^*^	**−1.093** ^*^	−0.553
Age	**−0.399**	−0.302	−0.312	−0.119
Marital status	0.323	0.163	0.119	0.300
Education	0.167	0.171	0.150	0.081
Professional title	−0.135	−0.229	−0.246	−0.152
Employment type	0.041	0.097	0.069	0.027
Department	−0.048	**−0.054**	−0.041	−0.026
Position	**−0.617** ^*^	**−0.702** ^*^	**−0.470** ^*^	**−0.398** ^*^
Monthly income	−0.037	−0.103	−0.165	−0.138
**Independent variable**
Sickness presenteeism		**1.307** ^*^	**0.981** ^*^	**0.848** ^*^
**Mediating variables**
Fatigue			**0.461** ^*^	**0.248** ^*^
Job burnout				**0.087** ^*^
*R*^2^^†^	0.026	0.104	0.218	0.326
*F*^‡^	**8.925**	**34.226**	**74.981**	**119.173**
Δ*R*^2^^§^	0.026	0.077	0.114	0.108
Δ*F*^¶^	**8.925**	**255.034**	**432.581**	**473.460**

*coef, nonstandard regression coefficient*.

*Bold value for p < 0.05*.

* *Statistically significant differences in the variables after application of Bonferroni correction (p < 0.013)*.

† *R^2^ refers to the proportion of variations in the dependent variable explained by the variables in the regression equation*.

‡ *F is the value that evaluates whether the regression equation holds*.

*§ΔR^2^ represents the increased value of the proportion of variations in the dependent variable explained by the regression equation as new variables are entered into the model*.

*¶ΔF is the value that evaluates whether the regression equation changes significantly as new variables continue to be introduced into the model*.

### Mediation Analysis

**Figure 1:**
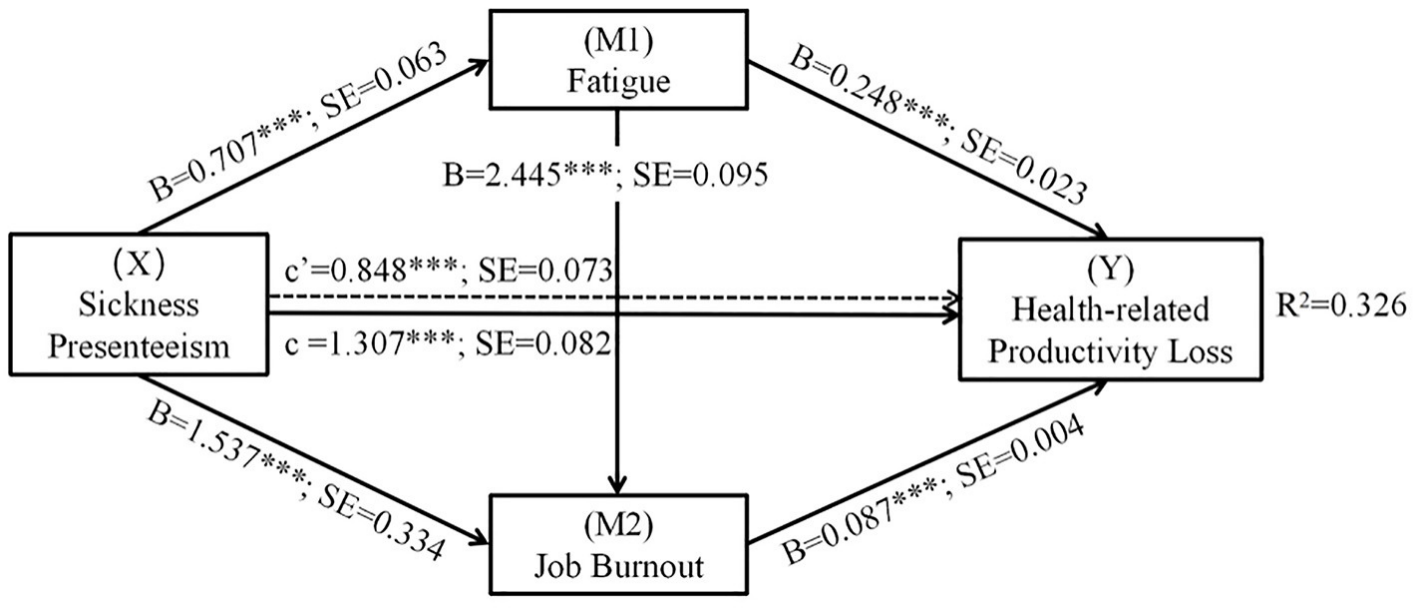
Serial-multiple mediating effect of fatigue and job burnout on the correlation between sickness presenteeism and health-related productivity loss. SE, standard error. B refers to the unstandardized path coefficients; X refers to the independent variable; M1 and M2 refer to mediating variables; Y refers to the dependent variable; Covariates include age, gender, marital status, education, professional title, employment type, department, position, and monthly income. ***p < 0.001.

According to Figure 1 and Table 5, a significant direct effect [c' = 0.848, standard error (SE) = 0.073, *t* = 11.656, *p* < 0.001] of sickness presenteeism on health-related productivity loss was observed. Significant direct paths from sickness presenteeism to fatigue (B = 0.707, *SE* = 0.063, *t* = 11.140, *p* < 0.001) and from fatigue to productivity loss (B = 0.248, *SE* = 0.023, *t* = 10.905, *p* < 0.001) were established. Statistically significant paths via the single mediation of fatigue [point estimate (PE) = 0.176; 95% bias corrected (BC) CI: 0.133–0.222] and job burnout (PE = 0.134; 95% BC CI: 0.073–0.193) were also established. Additionally, the paths from sickness presenteeism (B = 1.537, *SE* = 0.334, *t* = 4.605, *p* < 0.001) to job burnout and from job burnout (B = 0.087, *SE* = 0.004, *t* = 21.759, *p* < 0.001) to health-related productivity loss were of statistical significance. Finally, the path from fatigue (the first mediator) to job burnout (the second mediator) (B = 2.445, *SE* = 0.095, *t* = 25.807, *p* < 0.001) and that through both mediators (PE = 0.150; 95% BC CI: 0.119–0.184) were all of statistical significance.

**Table 5:** Comparison of indirect effects of sickness presenteeism on health-related productivity loss mediated by fatigue and job burnout (*N* = 2,968).

	**Product of coefficients**		**Bootstrapping 95% BC Cl**		**Percentage of total effect (%)**
	**Point estimates**	**Boot SE**	**Boot LL CI**	**Boot UL CI**	
**Model pathways**
Total effect: X → Y	1.307	0.082	1.147	1.468	100.00
Direct effect: X → Y	0.848	0.073	0.705	0.990	64.88
Total indirect effect: X → Y	0.459	0.046	0.369	0.549	35.12
Indirect effect 1: X → M1 → Y	0.176	0.023	0.133	0.222	13.47
Indirect effect 2: X → M2 → Y	0.134	0.030	0.073	0.193	10.25
Indirect effect 3: X → M1 → M2 → Y	0.150	0.017	0.119	0.184	11.48
**Contrasts**
Ind 1 minus Ind 2	0.042	0.038	**–**0.031	0.118	—
Ind 1 minus Ind 3	0.025	0.022	**–**0.017	0.068	—
Ind 2 minus Ind 3	**–**0.017	0.034	**–**0.083	0.049	—

*BC, bias corrected; CI, confidence interval; SE, standard error; LL, lower level; UL, upper level; Ind, indirect effect*.

*Number of bootstrapped samples for BC CI: 5,000*.

*Level of confidence for all CIs: 95%*.

*X, presenteeism; M1, fatigue; M2, job burnout; Y, health-related productivity loss*.

When we entered sickness presenteeism and the two mediators into the model, we found a significant total effect of sickness presenteeism on productivity loss (c = 1.307, *SE* = 0.082, *t* = 15.970, *p* < 0.001). Taken together, our findings revealed a serial-multiple mediating effect and a statistically significant total indirect effect (PE = 0.459; 95% BC CI: 0.369–0.549) that accounted for 35.12% of the total effect size. Additionally, three pairs of contrasting results were observed. There was no statistical difference in the power of mediating effect among the paths through single mediation by fatigue and job burnout and the path through serial-multiple mediation, which accounted for 13.47, 10.25, and 11.48% of the total effect, respectively.

## Discussion

### Relationship Between Sickness Presenteeism and Health-Related Productivity

Our data indicated that the incidence of sickness presenteeism in Chinese nurses was 70.6%, which is similar to the 74% reported in Saudi Arabia (37). This incidence of sickness presenteeism is markedly higher than those observed in Croatia (6.82%) (38), Korea (23.2%) (39), and the USA (42.1%) (40). Some possible reasons for this discrepancy were as follows. First, according to the statistical report released by the National Bureau of Statistics of China in 2020, the number of registered nurses in China was 4.4 million in 2019 and the number of nurses was 3/1,000 of the total population, a ratio that is much lower than western countries (41). The shortage of nursing human resources may lead to the sickness presenteeism of nurses (5). Second, the disparity in incidence reporting may be due to the different sickness presenteeism measurement tools used in diverse studies. For example, some studies chose the single-item sickness presenteeism questionnaire as used in this study (37, 39), the World Health Organization's health and work performance questionnaire (38), or created a self-made questionnaire (40). Third, nurses' workload increased significantly due to the need for vaccination, nucleic acid collection, and epidemiological investigations for COVID-19 prevention and control. An overwhelming workload is harmful to nurses' physical and mental health. However, nursing is believed to be an altruistic profession in Chinese culture, and working with sickness is considered an honorable and noble act (42).

The study revealed a strong positive correlation between sickness presenteeism and productivity loss due to health issues in nurses from China, which is consistent with the finding of a previous study (7, 17–19). Previous investigations also demonstrated that sickness conditions were a positive predictor of productivity loss in nurses (7, 43). This suggests that the more frequently nurses continue to work with sickness, the greater the total health-related productivity loss. Therefore, sickness presenteeism in clinical nursing work should not be encouraged, and ensuring nurses' health-related productivity should be the priority of nursing management.

Demographic characteristics such as age, employment type, and nurses' position were significantly associated with productivity loss in the univariate analysis. Nurses aged <40 years experienced a greater health-related productivity loss than nurses aged ≥40 years, which supported the previous result (44). Due to the current work environment, permanent staff nurses tended to have less occupational competitive pressure and better work benefits than other types of nurses (45). Employment disparity was highlighted in areas such as labor relations, social security, work assessment, and other aspects (45). Therefore, when health affects work, permanent staff nurses might be more effective in reducing the degree of health productivity loss. It is worth noting that the health-related productivity loss of clinical nurses was greater than head nurses. This could be because clinical nurses undertake work that requires more physical and mental strength, and meticulous clinical work has higher requirements for nurses' physical and mental health (45).

### Mediating Role of Job Burnout and Fatigue

We then investigated job burnout as a mediator and found that nurses with more sickness presenteeism behaviors were prone to a higher level of job burnout. This in turn resulted in reduced health-related productivity. This finding is consistent with the results of previous studies, which suggested that sickness presenteeism behaviors would be positively correlated with employees' burnout (8, 27). In addition, several prospective high-quality investigations exhibited the occupational outcomes of job burnout, including its adverse effects on health-related productivity (46). Our examination of the relationship between job burnout and health-related productivity loss confirmed a significant association between them. The impact of job burnout highlighted the necessity of preventive interventions and early recognition of these health conditions to avoid productivity loss. In addition, demographic characteristics as identified in this study were significantly associated with job burnout in the univariate analysis. This provided a basis for us to ascribe job burnout among nurses with different characteristics.

We also investigated fatigue as an additional mediator and found nurses with more sickness presenteeism behaviors were prone to a higher level of physical and mental fatigue, which in turn resulted in more health-related productivity loss. Employees who work with sickness tend to feel tired, have low work morale and a negative opinion of the job, which collectively resulted in impaired work productivity and poor work quality (9, 38). Based on a study by Lee et al., fatigue was one of the most important health conditions that induced productivity loss (43). Moreover, Espahbodi et al. (47) confirmed that sickness presenteeism and work productivity loss were both associated with higher fatigue levels.

Finally, to clarify the potential mechanism of fatigue and job burnout underpinning the correlation between sickness presenteeism and health-related productivity loss, we proposed a serial-multiple mediation model. We concluded that fatigue may result in job burnout, so sickness presenteeism is sequentially correlated with fatigue and job burnout, which may result in health-related productivity loss. Therefore, our result was somewhat supported by a previous study stating that physical and mental fatigue were positively correlated with job burnout (24). Our study confirmed that sickness presenteeism may increase or induce mental and physical fatigue among nurses, reduce their job engagement and job satisfaction, and increase job burnout (15). In addition, sickness presenteeism may also induce future physical and mental health problems, and impose a direct and indirect financial burden on health-care institutions through health-related productivity losses (16), which supported our conclusions.

Since nurses play an important role in universal healthcare, clarifying the mechanisms and links between nurse sickness presenteeism and health-related productivity loss is important. These determiners have profound implications for promoting public health and improving the overall quality of health care in the country (13, 20). This finding provides a reference to establish work health regulations, especially those on sickness presenteeism, and health-related productivity loss theories. Our study also suggests possible interventions that reduce health-related productivity loss among nurses could be achieved by decreasing sickness presenteeism, relieving fatigue, and reducing job burnout. Similarly, the reduction of sickness presenteeism and fatigue relief can help reduce job burnout.

### Limitations

First, the cross-sectional nature of this analysis hindered the establishment of a causal model to explain the correlations investigated in this study. Therefore, some caution should be exercised when interpreting the findings. Further investigations adopting experimental or longitudinal models are warranted to verify our conclusions. Second, this study only included hospitals in Shandong Province, China, thus limiting the generalization of the findings to other regions. Third, all measurement indexes were subjective data, which should be combined with economic indexes to objectively describe the loss of productivity. Fourth, since we adopted self-reported measures such as the instrument of sickness presenteeism in this study, response biases may have been inevitably introduced. Finally, the use of certain medications and the presence of previous psychopathology may affect the variables' assessment. Despite these limitations, to our knowledge, this is the first time researchers have explored the role of job burnout and fatigue between sickness presenteeism and health-related productivity loss among Chinese nurses using a serial-multiple mediation model.

## Conclusion

The incidence of sickness presenteeism among Chinese nurses was quite high. Additionally, demographic characteristics such as age, gender, education, professional title, employment type, department, position, and monthly income of nurses were significantly associated with sickness presenteeism, job burnout, fatigue, and productivity loss. Our findings also revealed that the high frequency of sickness presenteeism, after controlling for demographic variables, may result in increased productivity loss through the two mediating effects of fatigue and job burnout. In addition, sickness presenteeism may first increase fatigue, then promote job burnout, and finally, result in increased productivity loss among Chinese nurses.

## Data Availability Statement

The datasets presented in this article are not readily available because the data from the Nurses' Health Cohort Study of Shandong needs time for data clearing and establishment of guidelines. We are planning on opening this data to the public in the future. Requests to access the datasets should be directed to caoyj@sdu.edu.cn.

## Ethics Statement

The studies involving human participants were reviewed and approved by the Ethics Committee of Scientific Research of Shandong University Qilu Hospital. The patients/participants provided their written informed consent to participate in this study.

## Author Contributions

YL, BG, and YW: methodology, formal analysis, data curation, software, writing—original draft, and visualization. RL and XL: writing—review and editing and project administration. XG, LL, and JL: investigation. YC: conceptualization, resources, supervision, project administration, and funding acquisition. All authors contributed to the article and approved the submitted version.

## Funding

This study was funded by the National Key Research and Development Program of China (Grant Number 2020YFC2003500). The funders had no role in study design, data collection and analysis, decision to publish, or preparation of the manuscript.

## Conflict of Interest

The authors declare that the research was conducted in the absence of any commercial or financial relationships that could be construed as a potential conflict of interest.

## Publisher's Note

All claims expressed in this article are solely those of the authors and do not necessarily represent those of their affiliated organizations, or those of the publisher, the editors and the reviewers. Any product that may be evaluated in this article, or claim that may be made by its manufacturer, is not guaranteed or endorsed by the publisher.
