# Supplementary material for: Serial-Multiple Mediation of Job Burnout and Fatigue in the Relationship Between Sickness Presenteeism and Productivity Loss in Nurses: A Multicenter Cross-Sectional Study
Source: Front Public Health. 2022 Jan 14;9:812737. doi: 10.3389/fpubh.2021.812737 (PMC8795673; doi:10.3389/fpubh.2021.812737)
Supplement: Supplementary file 1 [file Table_1.DOCX]

Supplementary Material

# Supplementary Table 1 Univariate analysis of demographic factors related to sickness presenteeism in nurses.

| Variables | n (%) | Sickness presenteeism (x±SD) | t/F value^†^ | p-value |
| --- | --- | --- | --- | --- |
| Total | 2,968 (100.00) | 2.19±0.97 |  |  |
| **Gender** |  |  |  |  |
| Male | 137 (4.62) | 2.20±1.02 | 0.201 | <0.885 |
| Female | 2,831 (95.38) | 2.19±0.97 |  |  |
| **Age, years** |  |  |  |  |
| <30 | 745 (25.10) | 2.10±0.96 | **10.362** | **<0.001*** |
| 30-39 | 1,652 (55.66) | 2.19±0.95 |  |  |
| 40-49 | 460 (15.50) | 2.40±1.00 |  |  |
| ≥50 | 111 (3.74) | 2.01±1.07 |  |  |
| **Marital status** |  |  |  |  |
| Unmarried | 637 (21.46) | 2.06±0.98 | **5.273** | **0.001*** |
| Married | 2,273 (76.58) | 2.22±0.96 |  |  |
| Divorced | 39 (1.31) | 2.31±0.98 |  |  |
| Others | 19 (0.64) | 2.47±0.91 |  |  |
| **Education^‡^** |  |  |  |  |
| Secondary vocational degree | 789 (26.58) | 2.23±0.97 | 0.643 | 0.587 |
| Associate's degree | 1,613 (54.35) | 2.18±0.98 |  |  |
| Bachelor's degree | 557 (18.77) | 2.17±0.88 |  |  |
| Master's degree | 9 (0.30) | 2.44±0.88 |  |  |
| **Professional title** |  |  |  |  |
| Junior | 1,579 (53.20) | 2.10±0.95 | **10.814** | **<0.001*** |
| Intermediate | 1,198 (40.36) | 2.31±0.97 |  |  |
| Assistant senior | 184 (6.20) | 2.21±1.03 |  |  |
| Senior | 7 (0.24) | 2.00±1.16 |  |  |
| **Employment type** |  |  |  |  |
| Permanent staff | 886 (29.85) | 2.31±1.01 | **4.558** | **<0.001*** |
| Personnel agency | 1,534 (51.68) | 2.16±0.96 |  |  |
| Contract staff | 356 (11.99) | 2.09±0.88 |  |  |
| Labor dispatch | 133 (4.48) | 2.11±0.96 |  |  |
| Filing staff | 38 (1.28) | 2.37±1.00 |  |  |
| Others | 21 (0.71) | 1.71±0.90 |  |  |
| **Department** |  |  |  |  |
| Internal medicine | 849 (28.61) | 2.20±0.97 | 1.508 | 0.130 |
| Surgery | 624 (21.02) | 2.18±0.93 |  |  |
| Emergency | 183 (6.17) | 2.09±0.91 |  |  |
| Gynecology | 76 (2.56) | 2.18±0.98 |  |  |
| Obstetrics | 144 (4.85) | 2.05±0.96 |  |  |
| Pediatrics | 264 (8.89) | 2.12±0.99 |  |  |
| Operating room | 235 (7.92) | 2.34±1.01 |  |  |
| ICU | 175 (5.90) | 2.23±1.03 |  |  |
| Outpatient | 87 (2.93) | 2.36±1.02 |  |  |
| Administration | 6 (0.20) | 2.33±0.82 |  |  |
| Others | 325 (10.95) | 2.22±0.98 |  |  |
| **Position** |  |  |  |  |
| Clinical nurse | 2,620 (88.27) | 2.17±0.96 | **3.683** | **0.003*** |
| Deputy head nurse | 150 (5.05) | 2.37±1.01 |  |  |
| Head nurse | 185 (6.23) | 2.36±1.04 |  |  |
| General head nurse | 4 (0.13) | 2.00±1.41 |  |  |
| Deputy director of nursing department | 5 (0.17) | 2.60±0.55 |  |  |
| Director of nursing department | 4 (0.13) | 3.25±0.50 |  |  |
| **Monthly income, CNY** |  |  |  |  |
| <3,000 | 195 (6.57) | 1.97±0.91 | **6.244** | **<0.001*** |
| 3,000—5,999 | 1,504 (50.67) | 2.15±0.95 |  |  |
| 6,000—8,999 | 944 (31.81) | 2.25±0.98 |  |  |
| 9,000—19,999 | 280 (9.43) | 2.36±1.03 |  |  |
| ≥12,000 | 45 (1.52) | 2.09±1.08 |  |  |

Abbreviations: SD, standard deviation; ICU, intensive care unit; CNY, China Yuan.

Bold value for p < 0.05.

*Statistically significant differences in the variables after application of Bonferroni correction (p < 0.006).

^†^One-way ANOVA was carried out for more than two groups, and independent-samples t-test was adopted for two groups.

^‡^Secondary vocational degree: Having a 4-year senior high school study experience of professional training; associate's degree: Having a 3-year college study experience of professional training; bachelor's degree: Having a 4-year or 5-year undergraduate course of training.

## 
