# Supplementary material for: Serial-Multiple Mediation of Job Burnout and Fatigue in the Relationship Between Sickness Presenteeism and Productivity Loss in Nurses: A Multicenter Cross-Sectional Study
Source: Front Public Health. 2022 Jan 14;9:812737. doi: 10.3389/fpubh.2021.812737 (PMC8795673; doi:10.3389/fpubh.2021.812737)
Supplement: Supplementary file 2 [file Table_2.DOCX]

Supplementary Material

## Supplementary Table 2 Univariate analysis of demographic factors related to fatigue in nurses.

| Variables | n (%) | Fatigue (x±SD) | t/F value^†^ | p-value |
| --- | --- | --- | --- | --- |
| Total | 2,968 (100.00) | 8.48±3.40 |  |  |
| **Gender** |  |  |  |  |
| Male | 137 (4.62) | 8.67±3.41 | 0.479 | 0.489 |
| Female | 2,831 (95.38) | 8.47±3.40 |  |  |
| **Age, years** |  |  |  |  |
| <30 | 745 (25.10) | 8.17±3.50 | **9.360** | **<0.001*** |
| 30-39 | 1,652 (55.66) | 8.76±3.28 |  |  |
| 40-49 | 460 (15.50) | 8.09±3.53 |  |  |
| ≥50 | 111 (3.74) | 7.83±3.62 |  |  |
| **Marital status** |  |  |  |  |
| Unmarried | 637 (21.46) | 8.30±3.46 | 1.097 | 0.349 |
| Married | 2,273 (76.58) | 8.53±3.39 |  |  |
| Divorced | 39 (1.31) | 8.59±3.27 |  |  |
| Others | 19 (0.64) | 7.68±3.53 |  |  |
| **Education^‡^** |  |  |  |  |
| Secondary vocational degree | 789 (26.58) | 8.46±3.30 | 0.989 | 0.397 |
| Associate's degree | 1,613 (54.35) | 8.42±3.48 |  |  |
| Bachelor's degree | 557 (18.77) | 8.62±3.32 |  |  |
| Master's degree | 9 (0.30) | 9.89±2.52 |  |  |
| **Professional title** |  |  |  |  |
| Junior | 1,579 (53.20) | 8.43±3.43 | **3.471** | **<0.015** |
| Intermediate | 1,198 (40.36) | 8.64±3.32 |  |  |
| Assistant senior | 184 (6.20) | 7.86±3.56 |  |  |
| Senior | 7 (0.24) | 7.00±3.65 |  |  |
| **Employment type** |  |  |  |  |
| Permanent staff | 886 (29.85) | 8.37±3.42 | **3.287** | **0.006** |
| Personnel agency | 1,534 (51.68) | 8.45±3.40 |  |  |
| Contract staff | 356 (11.99) | 8.81±3.32 |  |  |
| Labor dispatch | 133 (4.48) | 8.93±3.41 |  |  |
| Filing staff | 38 (1.28) | 8.26±3.33 |  |  |
| Others | 21 (0.71) | 6.19±3.56 |  |  |
| **Department** |  |  |  |  |
| Internal medicine | 849 (28.61) | 8.60±3.29 | 1.259 | 0.248 |
| Surgery | 624 (21.02) | 8.41±3.46 |  |  |
| Emergency | 183 (6.17) | 8.72±3.34 |  |  |
| Gynecology | 76 (2.56) | 8.20±3.39 |  |  |
| Obstetrics | 144 (4.85) | 8.03±3.60 |  |  |
| Pediatrics | 264 (8.89) | 8.83±3.34 |  |  |
| Operating room | 235 (7.92) | 8.17±3.56 |  |  |
| ICU | 175 (5.90) | 8.66±3.41 |  |  |
| Outpatient | 87 (2.93) | 8.14±3.65 |  |  |
| Administration | 6 (0.20) | 7.50±3.45 |  |  |
| Others | 325 (10.95) | 8.33±3.36 |  |  |
| **Position** |  |  |  |  |
| Clinical nurse | 2,620 (88.27) | 8.55±3.38 | **3.794** | **0.002*** |
| Deputy head nurse | 150 (5.05) | 8.34±3.51 |  |  |
| Head nurse | 185 (6.23) | 7.62±3.53 |  |  |
| General head nurse | 4 (0.13) | 10.75±2.63 |  |  |
| Deputy director of nursing department | 5 (0.17) | 5.40±3.51 |  |  |
| Director of nursing department | 4 (0.13) | 8.50±4.44 |  |  |
| **Monthly income, CNY** |  |  |  |  |
| <3,000 | 195 (6.57) | 8.30±3.48 | 0.857 | 0.489 |
| 3,000—5,999 | 1,504 (50.67) | 8.42±3.44 |  |  |
| 6,000—8,999 | 944 (31.81) | 8.54±3.42 |  |  |
| 9,000—19,999 | 280 (9.43) | 8.74±3.09 |  |  |
| ≥12,000 | 45 (1.52) | 8.16±3.20 |  |  |

Abbreviations: SD, standard deviation; ICU, intensive care unit; CNY, China Yuan.

Bold value for p < 0.05.

*Statistically significant differences in the variables after application of Bonferroni correction (p < 0.006).

^†^One-way ANOVA was carried out for more than two groups, and independent-samples t-test was adopted for two groups.

^‡^Secondary vocational degree: Having a 4-year senior high school study experience of professional training; associate's degree: Having a 3-year college study experience of professional training; bachelor's degree: Having a 4-year or 5-year undergraduate course of training.
