# Supplementary material for: Serial-Multiple Mediation of Job Burnout and Fatigue in the Relationship Between Sickness Presenteeism and Productivity Loss in Nurses: A Multicenter Cross-Sectional Study
Source: Front Public Health. 2022 Jan 14;9:812737. doi: 10.3389/fpubh.2021.812737 (PMC8795673; doi:10.3389/fpubh.2021.812737)
Supplement: Supplementary file 3 [file Table_3.DOCX]

Supplementary Material

## **Supplementary Table 3** Univariate analysis of demographic factors related to productivity loss in nurses.

| Variables | n (%) | Productivity loss (x±SD) | t/F value^†^ | p-value |
| --- | --- | --- | --- | --- |
| Total | 2,968 (100.00) | 15.05±4.52 |  |  |
| **Gender** |  |  |  |  |
| Male | 137 (4.62) | 16.41±4.34 | **12.924** | **<0.001*** |
| Female | 2,831 (95.38) | 14.99±4.52 |  |  |
| **Age, years** |  |  |  |  |
| <30 | 745 (25.10) | 15.57±4.29 | **16.574** | **<0.001*** |
| 30-39 | 1,652 (55.66) | 15.19±4.51 |  |  |
| 40-49 | 460 (15.50) | 14.21±4.74 |  |  |
| ≥50 | 111 (3.74) | 13.05±4.50 |  |  |
| **Marital status** |  |  |  |  |
| Unmarried | 637 (21.46) | 15.35±4.28 | 1.766 | 0.151 |
| Married | 2,273 (76.58) | 14.98±4.58 |  |  |
| Divorced | 39 (1.31) | 15.18±4.07 |  |  |
| Others | 19 (0.64) | 13.63±5.59 |  |  |
| **Education^‡^** |  |  |  |  |
| Secondary vocational degree | 789 (26.58) | 14.53±4.55 | **5.468** | **0.001*** |
| Associate's degree | 1,613 (54.35) | 15.17±4.47 |  |  |
| Bachelor's degree | 557 (18.77) | 15.41±4.60 |  |  |
| Master's degree | 9 (0.30) | 16.78±2.99 |  |  |
| **Professional title** |  |  |  |  |
| Junior | 1,579 (53.20) | 15.39±4.44 | **15.532** | **<0.001*** |
| Intermediate | 1,198 (40.36) | 14.93±4.56 |  |  |
| Assistant senior | 184 (6.20) | 13.10±4.43 |  |  |
| Senior | 7 (0.24) | 12.43±4.50 |  |  |
| **Employment type** |  |  |  |  |
| Permanent staff | 886 (29.85) | 14.54±4.58 | **4.558** | **<0.001*** |
| Personnel agency | 1,534 (51.68) | 15.19±4.49 |  |  |
| Contract staff | 356 (11.99) | 15.37±4.70 |  |  |
| Labor dispatch | 133 (4.48) | 15.95±3.99 |  |  |
| Filing staff | 38 (1.28) | 16.00±3.19 |  |  |
| Others | 21 (0.71) | 14.10±4.59 |  |  |
| **Department** |  |  |  |  |
| Internal medicine | 849 (28.61) | 15.14±4.49 | **4.013** | **<0.001*** |
| Surgery | 624 (21.02) | 15.24±4.41 |  |  |
| Emergency | 183 (6.17) | 15.54±4.24 |  |  |
| Gynecology | 76 (2.56) | 13.95±4.89 |  |  |
| Obstetrics | 144 (4.85) | 14.53±4.59 |  |  |
| Pediatrics | 264 (8.89) | 14.92±4.58 |  |  |
| Operating room | 235 (7.92) | 15.56±4.60 |  |  |
| ICU | 175 (5.90) | 15.86±4.33 |  |  |
| Outpatient | 87 (2.93) | 13.17±5.01 |  |  |
| Administration | 6 (0.20) | 12.33±3.44 |  |  |
| Others | 325 (10.95) | 14.56±4.53 |  |  |
| **Position** |  |  |  |  |
| Clinical nurse | 2,620 (88.27) | 15.26±4.51 | **10.401** | **<0.001*** |
| Deputy head nurse | 150 (5.05) | 13.79±4.12 |  |  |
| Head nurse | 185 (6.23) | 13.24±4.48 |  |  |
| General head nurse | 4 (0.13) | 13.25±2.99 |  |  |
| Deputy director of nursing department | 5 (0.17) | 11.40±1.82 |  |  |
| Director of nursing department | 4 (0.13) | 15.25±6.60 |  |  |
| **Monthly income, CNY** |  |  |  |  |
| <3,000 | 195 (6.57) | 15.69±4.11 | **2.724** | **0.027** |
| 3,000—5,999 | 1,504 (50.67) | 15.20±4.51 |  |  |
| 6,000—8,999 | 944 (31.81) | 14.84±4.59 |  |  |
| 9,000—19,999 | 280 (9.43) | 14.59±4.65 |  |  |
| ≥12,000 | 45 (1.52) | 14.64±4.16 |  |  |

Abbreviations: SD, standard deviation; ICU, intensive care unit; CNY, China Yuan.

Bold value for p < 0.05.

*Statistically significant differences in the variables after application of Bonferroni correction (p < 0.006).

^†^One-way ANOVA was carried out for more than two groups, and independent-samples t-test was adopted for two groups.

^‡^Secondary vocational degree: Having a 4-year senior high school study experience of professional training; associate's degree: Having a 3-year college study experience of professional training; bachelor's degree: Having a 4-year or 5-year undergraduate course of training.
